# Quality of Honey Imported into the United Arab Emirates

**DOI:** 10.3390/foods12040729

**Published:** 2023-02-07

**Authors:** Tareq M. Osaili, Wael A. M. Bani Odeh, Maryam S. Al Sallagi, Ahmed A. S. A. Al Ali, Reyad S. Obaid, Vaidehi Garimella, Fatema Saeed Bin Bakhit, Hayder Hasan, Richard Holley, Nada El Darra

**Affiliations:** 1 Department of Clinical Nutrition and Dietetics, College of Health Sciences, University of Sharjah, Sharjah P.O. Box 27272, United Arab Emirates; 2 Department of Nutrition and Food Technology, Faculty of Agriculture, Jordan University of Science and Technology, P.O. Box 3030, Irbid 22110, Jordan; 3 Studies and Risk Assessment Unit, Dubai Municipality, Dubai P.O. Box 67, United Arab Emirates; 4 Food Studies and Policies Section, Dubai Municipality, Dubai P.O. Box 67, United Arab Emirates; 5 Dubai Central Laboratories Department, Dubai Municipality, Dubai P.O. Box 67, United Arab Emirates; 6 Department of Food Science and Human Nutrition, University of Manitoba, Winnipeg, MB R3T 2N2, Canada; 7 Department of Nutrition and Dietetics, Faculty of Health Sciences, Beirut Arab University, Tarik El Jedidah—Beirut, P.O. Box 115020, Riad El Solh, Beirut 1107 2809, Lebanon

**Keywords:** honey, adulteration, non-compliant quality, hydroxymethylfurfural, sugar, imported

## Abstract

This study was performed to assess the physicochemical quality characteristics of honey imported by the United Arab Emirates (UAE) via Dubai ports between 2017 and 2021. There were 1330 samples analyzed for sugar components, moisture, hydroxymethylfurfural (HMF) content, free acidity, and diastase number. Of the honey tested, 1054 samples complied with the Emirates honey standard, but 276 (20.8%) did not; this was due to non-compliance with one or more quality parameters, thus suggesting some level of adulteration, improper storage or inappropriate heat treatment. For the non-compliant samples, the average values of sucrose content ranged from 5.1 to 33.4%; the sum of glucose and fructose ranged from 19.6 to 88.1%; the moisture content varied from 17.2 to 24.6%; the HMF occurred in a range from 83.2 to 663.0 mg/kg, and the acidity varied from 52 to 85 meq/kg. The non-compliant honey samples were grouped according to their country of origin. India was shown to be the country having the highest percentage of non-compliant samples at 32.5% and Germany had the lowest at 4.5%. This study emphasized that the inspection of honey samples traded internationally should involve physicochemical analysis. A comprehensive inspection of honey at the Dubai ports should reduce incidents of adulterated products being imported.

## 1. Introduction

Honey is an ancient natural product valued for its health benefits, medical characteristics and biological properties [1,2]. According to the definition of Codex Alimentarius [3] and a European Community Directive [4], honey is a natural substance produced by honeybees from the nectar of flowers without any added ingredients. Usually, the quality of honey is assessed by several parameters, including its moisture, sugar content, pH, total acidity, hydroxymethylfurfural (HMF) content and other factors [3,5,6]. The sweet taste of honey is primarily due to its high fructose content [4,6]. Consumers generally perceive it as a natural and healthier sweetening alternative to table sugar [7].

Due to its high price, honey is frequently adulterated [8]. It is ranked sixth amongst the food products subjected to fraud in Europe [9]. Therefore, strict monitoring and quality assurance are needed to prevent adulterated products from entering the consumer market. Adulteration is only one practice by which the quality of honey can be compromised. Other factors include, but are not limited to, the application of a heat treatment or the use of improper storage conditions [10,11].

Due to the high variability in honey composition, the detection of adulteration is not an easy task. Honey adulteration can be carried out by adding water (dilution) or cheap sugar solutions, such as high-fructose corn syrup [12]. High-quality honey can also be mixed with honey of low quality, and sold at a higher price [13]. Adulteration may compromise consumer experience and expectations, which may lead to the reduced demand for honey and its products. Moreover, legitimate honey producers are often unable to compete with the low-priced adulterated honey. Consequently, there is growing interest in screening honey for adulteration and quality before market distribution.

Various methods have been developed to check for honey adulteration, with each method having its advantages [14]. Physicochemical analysis, such as of the sucrose content, the sum of glucose and fructose, moisture content, HMF, acidity, or diastase activity, are often used for this purpose [12,15]. Honey may be considered adulterated if one or more of these parameters does not meet international or domestic standards. Analysis of the sucrose content is also often used to identify honey adulteration. Authentic honey must not contain more than 5% sucrose [16]. Honey is mainly composed of glucose and fructose in varying concentrations (55 to 75 g/100 g), with a minimum acceptable limit of 60%. The second main component of honey is water (15 to 25 g/100 g), with an acceptable moisture level being below 17% [17]. HMF is an indicator of honey freshness and is commonly used in quality analysis. It is a compound formed during the acid-catalyzed dehydration of hexoses [18]. Generally, it should be present in very low amounts in fresh honey, with its complete absence being indicative of high-quality honey. As per the Codex Alimentarius honey standard [3] and the United Arab Emirates (UAE) standard, HMF content must not be higher than 80 mg/kg. The EC Council directive 2001/110/EC from the European community has set an HMF limit of 40 mg/kg, with an exceptional value of 80 mg/kg for honey coming from countries with tropical temperatures [4]. Honey with an HMF value of >80 mg/kg is characterized as being of very low quality. HMF quantities are affected by pH, heat treatment [19], and the conditions used for honey storage [20], with warm environments increasing the HMF concentration [21]. In addition, mixing honey with invert syrups can also increase HMF values [22]. Honey contains small amounts of different enzymes, and one of the most important ones is amylase (diastase). This enzyme is sensitive to heat and is, therefore, able to indicate the overheating of honey and its degree of thermal preservation [23]. Thus, diastase activity is considered to be a quality indicator used for the freshness of honey, set by Codex [3]. The diastase activity is usually expressed in Schade units, also known as the diastase number (DN), which is defined as the amount of enzyme that will convert 0.01 g of starch to the prescribed end-point in 1 h at 40 °C under the conditions of the test [24]. According to the Honey Quality and International Regulatory Standards, the diastase activity must not be less than or equal to eight, determined after processing and blending for all retail honey.

Besides the above stated parameters, free acidity is also used to identify fraudulent honey. The Codex Alimentarius [3] has set a permitted range of 50 meq acid/kg. A high value of free acidity in honey is an indicator that glucose and fructose fermentation by yeasts has occurred, converting the sugars to alcohol and carbon dioxide. In the presence of oxygen, alcohol is hydrolyzed to acetic acid, consequently increasing the free acidity [21]. On an obvious note, physical contaminants, such as hair and insects, are unacceptable. Recent research work has evaluated the quality of honey. A study by Kazeminia, Mahmoudi, Aali, and Ghajarbygi (2021) [25] assessed 43 honey samples collected from Qazvin province, Iran, and showed that the pH and acidity values conformed perfectly (100%) with the Iranian honey standard. However, 44.2% of the samples did not meet the acceptable quality level regarding HMF. For moisture content, 2.3% of the samples were above the acceptable limit. There was also a high percentage of samples that were not compliant with sucrose (53.5%), and glucose and fructose (25.6%) content requirements. Another study conducted by Gürbüz et al. (2020) [26] reported that all 68 honey samples collected from the Southeastern Anatolia region of Turkey were in compliance with the international standard in Turkey for sucrose content, the sum of glucose and fructose, moisture content and free acidity. However, 20.6% of samples were non-compliant concerning diastase number. For HMF, 7 of 68, or 10.3% of honey samples, had a higher HMF content than the legally permitted EU maximum level of 40 mg/kg. A study conducted by Yayinie, Atlabachew, Tesfaye, Hilluf, and Reta (2021) [27] found that all 47 honey samples collected from different geographical areas within the Amhara region, Ethiopia, met the Codex Alimentarius [3] standard. Boussaid et al. (2018) [28] reported that all 9 honey samples collected from southern Tunisia met the standards of the Codex Alimentarius for pH, free acidity, water activity and HMF.

Since there has not been any investigation available that has examined the imported honey quality in the UAE, the present study was undertaken to assess the honey imported to the UAE via ports in the Dubai Emirates over a 5 year period.

## 2. Materials and Methods

### 2.1. Sample Collection

A total of 1330 honey samples were collected from Dubai ports in the UAE between 2017 and 2021. They were classified according to honey type: honey (1180), blended honey (58), honeycomb (47), acacia honey (25), and forest honey (20). Blended honey included honey mixed with spices, pollen, verbena, lemon, mint, chili, ginger, pepper, cinnamon, or hibiscus. Comb honey is produced from traditional hives [29] and it contains honeycomb, which is the wax structure in which honey bees store honey and pollen in these hives. Acacia honey is a monofloral honey produced by *Apis mellifera*, a cultured bee that harvests the extra-floral nectar from the forest mangrove or mangium tree (*Acacia mangium*) [30]. Forest honey is produced by bees from oak, holm oak, and cork oak forests.

The sampling of honey was conducted by trained, authorized food inspectors from 5 sites located in the Dubai ports, municipality of Dubai. The collected samples were sent to the food analysis laboratory where they were stirred to yield uniformity within each sample, and then analyzed.

### 2.2. Chemicals and Reagents

All the solvents and chemicals used in extraction procedures and in the preparation of mobile phases were of LC-MS/MS reagent grade and were obtained from Sigma-Aldrich Chemie GmbH (Taufkirchen, Germany). Milli-Q ultra-pure water was used for all analyses (Merck, Milli-Q^®^ IQ Element Water Purification, Burlington, MA, USA).

### 2.3. Determination of Sugar Composition

Determination of sugars (fructose, glucose, and sucrose) was carried out by High Performance Liquid Chromatography (Agilent Infinity 1260 II, Agilent, Santa Clara, CA, USA), using a refractive index detector following the AOAC official method 977.20-1977. Sample preparation was carried out by dissolving 2 mg honey in 25 mL deionized water. Standard solutions of 1% fructose, 1% glucose, and 0.5% sucrose (Sigma-Aldrich, St. Louis, MO, USA) were prepared in distilled water. Mixed standards were prepared at 1.0% for fructose and glucose and 0.5% for sucrose. The mixed standard was diluted to yield 0.2, 0.4, 0.6 and 0.8%. The chromatographic separation of sugars was achieved using an Agilent Zorbax carbohydrate column maintained at 35 °C with acetonitrile/water (75:25, *v*/*v*), which was used as the mobile phase. Then 10 µL of sample was injected at a flow rate of 1.0 mL/min. The temperature of the column was maintained at 27 °C during the entire run.

### 2.4. Moisture Analysis

The moisture content of the honey samples was determined using the refractive index (RI). Water content was obtained from a Chataway table [31]. An automatic digital refractometer, Atago RX-5000α (Bashumi Instr. Control Services, Northriding, Randburg, SA), calibrated with distilled water, was used for the measurement. A drop of honey was placed on the surface of the prism and a refractive index; the reading was taken at 20 °C and converted to a percentage (g/100 g) using the Chataway table [31].

### 2.5. Determination of Hydroxymethylfurfural (HMF)

The HMF content was determined based on the UV absorbance of HMF at 284 nm using a UV-Visible spectrophotometer (Thermo Fisher Scientific, Waltham, MA, USA) [32]. In order to avoid the interference of other components at this wavelength, the difference between the absorbance of a clear aqueous honey solution and the same solution after the addition of bisulfite was determined. The HMF content was calculated after the subtraction of the background absorbance at 336 nm. In a 50 mL volumetric flask containing 2 mg of honey dissolved in 25 mL of water, 0.5 mL of Carez I solution was added, followed by 0.5 mL of Carez II solution. Water was added to the flask to make up a volume of 50 mL, and the resulting solution was filtered. After discarding the first 5 mL of the filtrate, 5 mL of 0.2% sodium bisulfite solution was added to the test tube. In another test tube, 5 mL of pure water was added as a blank. A UV-visible spectrophotometer was used to measure the solution’s absorbance at 284 and 336 nm in 10 mm quartz cells within 1 h. The calculation was performed using the formula below:

$$\mathrm{HMF}\;(\mathrm{mg}/\mathrm{kg})=(A284-A336)\;\times \;149.7\;\times \;5\;\times \;\frac{D}{W}$$

It should be noted that 149.7 is a constant, A284 is the absorbance at 284 nm, A336 is the absorbance at 336 nm, 5 is the theoretical nominal sample weight, D is the dilution factor (in case dilution is necessary), and W is the weight of honey taken.

### 2.6. Determination of Honey Acidity

A titrimetric method was used to determine free acidity following the AOAC official method 962.19-1977. In a 100 mL beaker, a 3 mg sample of homogenized honey was dissolved in water. The solution was then titrated against a 0.1 N NaOH solution until the formation of a pink color and the titer value was noted. The results were reported as milliequivalents (meq) per kg of honey [33].

### 2.7. Diastase Activity

Diastase activity was calculated according to the AOAC method 958.09 [34]. The honey sample (5 g) was diluted in 10 mL of deionized water and 2.5 mL of acetate buffer (1.59 M, pH 5.3). The diluted sample was then transferred to a 25 mL volumetric flask containing 1.5 mL of 0.5 M NaCl solution. Ten ml of honey solution was mixed with 100 mL of 1% (*w*/*v*) starch solution and incubated in a water bath at 40 °C for 5 min. After that, 1 mL of the treated sample was mixed with 10 mL of 0.0007 M diluted iodine solution and measured at 660 nm in a spectrophotometer (Thermo-scientific Evolution 60S model, Waltham, MA, USA).

### 2.8. Physical Contaminants

The determination of extraneous matter was conducted by visual inspection. Honey samples were checked for the presence of hair, other foreign material, or insect parts.

## 3. Results and Discussion

### 3.1. Compliance of Imported Honey with Standards

The conformity assessment of imported honey samples, based on the UAE honey standard [35] in Table 1, is presented in Table 2. The data presented are arranged to identify the types of honey that are more likely to be adulterated. An evaluation of 1330 samples collected revealed that 79.2% conformed with UAE legislation. The level of non-conformity was lower than the 64.4% reported by Al-Farsi et al. (2018) [6] for 58 honey samples collected from 18 geographical regions in Oman, following a comparison with Gulf Standardization Organization (GSO) standards. It is notable that Boussaid et al. (2018) [28] found that all 9 honey samples examined in Tunisia complied with the Codex Alimentarius standards.

In the current study, the proportion of non-conformity ranged from 5.0 to 21.0% for the various types of honey, with the lowest values noted for forest honey. Honey, blended honey, and honeycomb had non-conformity levels that were similar, at 20.5 to 21.0%.

Table 3 presents the honey compliance across the years from 2017 to 2021. For the individual years, statistical significance was noted between the conforming and non-conforming samples. However, across the years, only 2017 was shown to be significantly different from the other years; here, 174, or 90.6%, of 1054 samples were compliant, while 18, or 9.4%, of 276 were non-compliant. None of the other years (2018, 2019, 2020 or 2021) had significantly different numbers of non-conforming samples, but in 2019, the proportion of conforming samples was the lowest.

### 3.2. Compliance of Imported Honey with Recognized Standards

The honey samples were assessed for compliance with the Emirates standard [35] for moisture, total sucrose content, the sum of glucose and fructose, HMF, acidity limit, diastase activity and the presence of physical contaminants (Table 4).

#### 3.2.1. Total Sugar Content in Honey Samples

##### Sucrose Content

In 2.4%, or 6/251 samples of honey, non-compliance with the UAE honey standard was only due to sucrose content being higher than the 5% limit permitted. This level of non-compliance was lower than the 53.5% non-conformity, due to the high sucrose found among the 43 honey samples that originated in Iran [25]. During another study conducted by Gürbüz et al. (2020), sucrose was not detected in 55.9% or 38/68 honey samples collected from the Southeastern Anatolia region of Turkey [26]. The sucrose content of the remaining 30 samples (44.1%) was less than the legally permissible maximum value of 5%. In other work involving 9 honey samples collected from southern Tunisia it was found that the sucrose content ranged from 2.3 to 4.5% [28]. In the present study, the mean sucrose content of the 6 non-conforming samples was 14.1%, with a range of 5.1 to 33.4%. This high amount of sucrose could have been due to overfeeding the bees with sugar in spring [37] or to the early harvesting of honey before the full transformation of sugar into glucose and fructose [38]. The high sucrose content could be also an indication of possible adulteration by the direct addition of sugar to honey [39].

##### Sum of Glucose and Fructose

The determination of reducing sugars (the sum of glucose and fructose) in honey is also a quality criterion used to indicate honey freshness. Of the total number of non-conforming samples (n = 276), 113 (40.9%) did not meet the UAE criterion for acceptable total sugar content. While all 20 forest honey samples met the UAE standard, blended honey, honeycomb and acacia honey did not. Honey showed the highest percentage of non-conformities at 40.2%. The mean total reducing sugar content of these samples was 52.5%, with a range of 19.6 to 59.1%. An amount of fructose and glucose below 60% is taken to indicate honey adulteration. It should be noted that the ratio of fructose to glucose in any particular honey depends largely on the source of the nectar [37]. The mean total glucose and fructose content obtained in the present study for non-conforming honey samples at 52.5% was similar to the 54.3% obtained for 29 samples of Sidr honey collected from Oman. The acceptable total glucose and fructose content as per GSO honey standards is a minimum of 45% [6]; however, the UAE legislation is more strict and accepts a minimum value of only 60%.

The sugar composition results found during the current study are not in accordance with the values reported in a study by Geană, Ciucure, Costinel, and Ionete (2020) [40] where the total amount of fructose and glucose in 48 honey samples that originated in Romania was higher than the 60% specified in the EU standards [4]. Another study conducted in Turkey showed that the mean combined glucose and fructose content of 68 honey samples ranged from 62.6 to 77.3%, with a mean concentration of 71.0% [26].

#### 3.2.2. Moisture Content

The non-conformity concerning the moisture content was 12.7%. The moisture content of the samples was assessed based on the sampling year, since the permissible moisture content in honey according to the UAE standard was recently increased from 17% [36] to 20% [35]. In honey samples, 26 of the 31 samples did not meet the old criterion for moisture (UAE.S 147, 2017) [36], with a mean moisture content of 18.3% (17.7 to 19.3%). Using the updated criterion of 20% moisture, 5 of the 31 samples did not conform [38], and had a mean moisture content of 22% (20.6 to 24.6%). Similar values were reported for Tanzanian honey [41], with a moisture range of 21.6 to 22.8%, and Philippine honey [41], with a moisture range between 22.0 to 23.1%. In contrast, Australian honey samples were fully compliant with the 20% requirement, having mean sample moisture contents ranging from 10.6 to 17.8% [21]. High moisture content may have resulted from honey harvest under high humidity conditions or an early seasonal honey extraction [42]. The variance in moisture content can also be attributed to the botanical source, the season, as well as the geographic conditions.

#### 3.2.3. HMF Content

HMF is commonly used as a parameter for honey freshness and authenticity. Normally, fresh honey contains low amounts of HMF, with the HMF content depending on the rate of honey monosaccharide decomposition. According to the UAE honey standard [35], HMF content should not exceed 80 mg/kg in honey from countries with tropical temperatures. Of the 276 samples that did not conform to the standards, 72, or 26.1%, had HMF values above 80 mg/kg. For blended honey, 5 of the 12 non-conforming samples had an average value of 236.4 mg/kg (107 to 458 mg/kg) of HMF. For honey samples, 67 of 251 non-compliant samples had a mean HMF content of 154.7 mg/kg (83.2 to 663 mg/kg). The results of the present study are in agreement with those from a study conducted by Gürbüz et al. (2020) [26], where it was found that 10.3% of the honey samples had a higher HMF content than the legally permitted maximum of 40 mg/kg. The results of the current study are also similar to those from a study conducted by Al-Farsi et al. (2018) [6], where the values observed were 16.2 and 1062 mg/kg in Sumer and multiflora honey, respectively. Ajlouni and Sujirapinyokul (2010) [21] observed that HMF in two Australian honey samples (2.22 and 17.7 mg/kg) was within the international limit of 40 mg/kg. The variation in HMF values could result from the influence of factors such as pH, heating, storage conditions and floral type [43]. High HMF values are a reflection of overheating honey, use of inappropriate storage conditions or its mixture with an invert syrup made by acid or enzymatic inversion [37].

#### 3.2.4. Acidity

The legislation of the UAE [35] and Codex regulations [3] do not accept an acidity of >50 meq/kg in honey. This criterion was not met by 2% of the samples in the current study. The free acidity in the studied honey samples was 74 meq/kg (52 to 85 meq/kg). Similarly, in Oman, mean acidity values of 84.9 meq/kg were reported for Sumer honey, with 18 of 21 samples exceeding the Codex standard [6]. Research studies on honey collected from Turkey [44], Portugal [45], Argentina [46], and Ethiopia [39] showed lower values of free acidity (25.0, 40.3, 11.0, and 45.0 meq/kg, respectively). The high acidity observed in 5 samples in the present study could have been due to the fermentation of honey sugars to form organic acid [30]. This could be controlled by adopting more modern techniques for producing honey that enable reducing the moisture or by pasteurizing the honey to control microorganisms. The variation in acidity between the samples may also be attributed to the presence of different acids of varying floral origin or harvest season [44,45].

#### 3.2.5. Diastase Activity

It is well known that the natural enzyme diastase is an indicator of freshness in honey. The activity of the enzyme can be a measure of honey exposure to heat and/or inappropriate storage conditions [15,46]. All the honey samples in the present study were observed to have diastase activity, which was in accord with UAE standards [35]. The minimum standard value for the diastase index observed was 8 in all the samples, except 1 (honey, diastase value = 2). It is probable that this sample was not fresh or was inadequately pasteurized. This is in contrast with results from a study conducted on honey in Oman, where 16 of 58 samples did not conform to the GSO standard. In Iran, Kazeminia et al. (2021) [25] observed that 46.5% of 43 honey samples were non-compliant with standards for diastase activity. Compliant values for diastase were reported for honey in Ethiopia [47] and Argentina [48].

#### 3.2.6. Physical Contaminants

As per UAE legislation, samples are required to be free from any form of physical contaminants. In the present study, only 5 of 251 honey samples did not meet the criterion. Two of them had hair, and 3 were found to contain insect parts. In a study conducted by Brasil da Silva (2021) [49] on 14 honey samples collected from the north of Brazil, it was reported, following microscopic examination, that 50% (7/14) of the samples contained what appeared to be dirt. The presence of physical contaminants could result from improper processing practices including poor hygiene, inadequate storage conditions, poor pest control practices, as well as inadequate packaging.

#### 3.2.7. Combination of Non-Conforming Elements

Of the 276 non-conforming honey samples, 39 (14.1%) had more than one instance of non-compliance with the quality criteria. Approximately 35/251 samples, or 13.9%, of the honey samples did not meet the quality criteria on more than one count, with the distribution being as follows: glucose, fructose, and sucrose (9 samples); glucose, fructose, and HMF (8 samples); HMF and moisture (8 samples); glucose, fructose and moisture (4 samples); glucose, fructose, HMF, and moisture (2 samples); glucose, fructose, and acidity (2 samples); sucrose and HMF (1 sample); and HMF, diastase content, and sucrose (1 sample).

The combination of non-conformities in 9 samples (glucose, fructose and sucrose) may have resulted from the addition of sucrose or table sugar, which will reduce the percent of glucose and fructose present in total sugars detected. The combination of non-conformities in one sample (HMF, diastase content and sucrose) was probably due to the exposure of this sample to overheating; published work has shown that an increased temperature increases HMF and reduces diastase activity, especially for temperatures over 60 °C [50]. Therefore, the sample that was non-compliant due to a combination of HMF and diastase levels was not acceptably fresh.

The majority of the combined infractions included the violation of glucose and fructose content, which is highly affected by climate, honeybee flora, and honey handling practices [51].

#### 3.2.8. Classification of Honey Non-Conformities According to the Country of Origin

Samples that did not conform with international and local standards, as per the country of origin, and honey type are presented in Table 5. Of the 251 non-conforming honey samples, 137, or 32.5% of samples, originated in India. This high percentage of non-conformity is in agreement with a report published by the Centre for Science and Environment, which indicated a high percentage of non-compliance in honey samples from India, with 77% identified as being adulterated with sugar syrups.

In total, 10 of the 13 honey brands failed the purity test used during a study by Dhingra (2020) [52]. However, it should be noted that the highest number of samples examined had been imported from India in the study because India greatly increased honey exports recently [53]. This could potentially introduce bias in the observations of the study. India exports honey to more than 65 countries. The United States is the biggest importer of honey, largely from India, with approximately 80% of the total imported honey being of Indian origin. The UAE is the third largest importer of honey from India with a value of USD 2.66 million, representing 3.31% of the total Indian exports [54]. In the current study, 33, or 19.5%, of 170 samples from Australia did not conform to the standards. This percentage of non-conformity is in accord with the 18% found during a study conducted by Zhou, Taylor, Salouros, and Prasad (2018) [55] on Australian honey samples. In the current study, 12 samples from New Zealand, 9 samples from Pakistan, and 8 from Turkey did not meet the legislative criteria. For blended honey, 5 of the 12 non-compliant samples originated from India. For honeycomb, out of the 10 non-conforming samples, 4 samples originated from Turkey.

**Figure 1:**
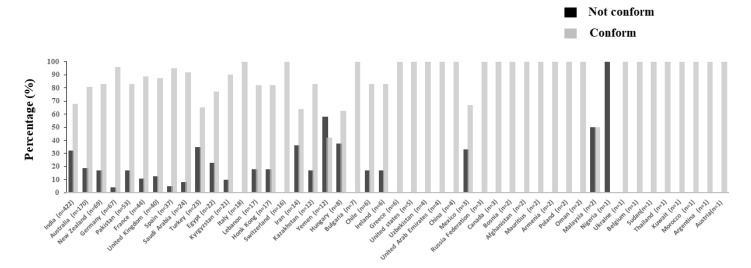
Origin and percent of imported honey samples (n = 1180) tested that were not compliant with UAE honey quality standards.

To better understand the results, a detailed conformity assessment is presented in Figure 1, according to the country of origin of honey samples (n = 1180). This approach was chosen only for the honey samples because of its greater sample size; it represented 88% of the total 1330 samples examined.

The honey samples originated from 5 of the 49 countries that supplied honey in this study, namely India (422), Australia (170), New Zealand (69), Germany (67), and Pakistan (53). India had the highest proportion of non-compliant samples (32.5%), while Germany had the lowest at 4.5%.

**Figure 2:**
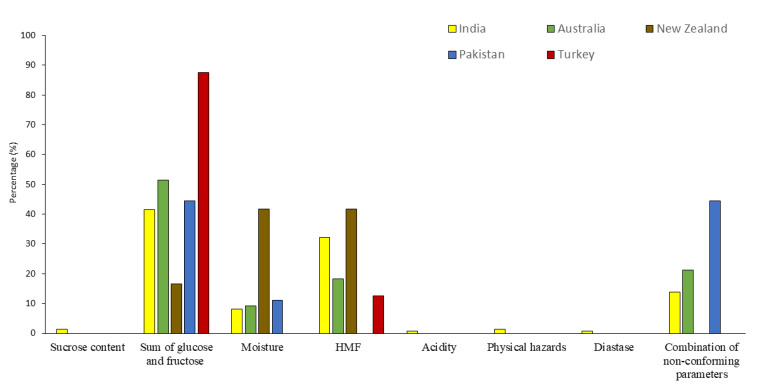
Occurrence of non-compliant parameters in imported honey samples from countries having the greatest levels of non-compliance.

The most frequent non-compliant parameters were the sum of glucose and fructose in samples originating from India, Australia, Pakistan, and Turkey (Figure 2). On the other hand, for samples from New Zealand, the moisture content and HMF were the major non-compliant parameters.

## 4. Conclusions

Assessment of the physicochemical parameters of honey is necessary for quality assurance purposes. This study evaluated the physicochemical quality characteristics of honey imported to the UAE. Of the 1330 honey samples tested, 1054 complied with the UAE honey standard. Examination of the country of origin showed that India supplied the highest proportion of non-compliant samples, at 32.5%, while Germany had the lowest, at 4.5%. However, it is important to note that the number of samples from each country differed and hence this may have introduced bias. The most frequent source of non-compliance involved the sum of glucose and fructose. This study emphasizes the need for continued border inspection, in conjunction with the physicochemical analysis of honey samples, in order to prevent the entry of adulterated honey into the country.

## Figures and Tables

**Table 1:** UAE standards of honey [35].

	UAE.S 147, 2019
Sucrose content	Max 5%
Sum of glucose and fructose	Min 60%
Moisture *	Max limit 20%
HMF	Max 80 mg/kg
Acidity	Max 50 meq/kg
Physical hazards (hair, insects)	Absent
Diastase activity	8 ⁰ Goth

* The permissible moisture content in honey according to the UAE standard was previously 17% [36].

**Table 2:** Summary of imported honey compliance with UAE standards from 2017 to 2021.

Type of Honey	No. Samples	No. Compliant (%)	No. Non-Compliant (%)
Honey	1180	929 (79)	251 (21)
Blended honey	58	46 (79.5)	12 (20.5)
Honeycomb	47	37 (79)	10 (21)
Acacia honey	25	23 (92)	2 (8)
Forest honey	20	19 (95)	1 (5)
Total	1330	1054 (79.3)	276 (20.8)

**Table 3:** Honey compliance across the years from 2017 to 2021 using Chi-square.

	Results		
	Conforming (n = 1054) n (%)	Non-Conforming (n = 276) n (%)	*p*-Value
2017 (n = 192)	174(90.6) ^aB^	18(9.4) ^bB^	<0.001
2018 (n = 268)	223(83.2) ^aA^	45(16.8) ^bA^	
2019 (n = 297)	201(67.7) ^aA^	96(32.3) ^bA^	
2020 (n = 263)	225(85.6) ^aA^	38(14.4) ^bA^	
2021 (n = 310)	231(74.5) ^aA^	79(25.5) ^bA^	

a, b: Different letters indicate significant differences in the proportions of conforming and non-conforming samples across the individual years (*p* < 0.001). A, B: Different letters indicate significant differences in the proportions of conforming and non-conforming samples across the different years (*p* < 0.001).

**Table 4:** Identification of non-compliant quality parameters among the 276 imported, non-compliant honey samples.

Type of Non-Compliant Honey		Sucrose Content (Max 5%)	Sum of Glucose and Fructose (Min 60%)	Moisture Max Limit		HMF (Max 80 mg/kg)	Acidity Max 50 meq/kg	Physical Hazards (Hair, Insects)	Diastase Activity (⁰ Goth)	Violations Involving Multiple Non-Compliant Parameters
				17%	20%					
Honeycomb (n = 10)	No. (%)		8 (80)	1 (10)						1 (10)
	Mean (%)		43.86	18.1						
	Range (%)		22.5−56.4	18−18.2						
Acacia honey (n = 2)	No. (%)		1 (50)	1 (50)						
	Mean (%)		50.4	18.6						
	Range (%)									
Blended honey (n = 12)	No. (%)		3 (25)	1 (8.)		5 (41.)				3 (25)
	Mean (%)		51.95	17.8		236.3				
	Range (%)		41.9−57.5			107−458				
Forest honey (n = 1)	No. (%)			1 (100)						
	Mean (%)			17.7						
	Range (%)									
Honey (n = 251)	No. (%)	6 (2.4)	101 (40.2)	26 (10.4)	5 (2)	67 (26.7%)	5 (2.0)	5 (2.0)	1 (0.4)	35 (13.9)
	Mean (%)	14.1	52.5	18.3	22	154.7	74	Present	2.1	
	Range (%)	5.1−33.4	19.6−59.1	17.7−19.3	20.6−24.6	83.2−663	52−85	Present		
Number of non-compliant samples (276)	No (%)	6 (2.2%)	113 (40.9)	35 (12.7%)		72 (26.1%)	5 (1.8%)	5 (1.8)	1 (0.4)	39 (14.1)

**Table 5:** Number of non-conforming samples (%) according to the country of origin and type of honey.

Country (No. of Imported Samples)	No. of Imported Samples (No. of Rejected Samples, %)				
	Honey	Blended Honey	Honey Comb	Acacia Honey	Forest Honey
India (430) ^$^	422 ^$$^ (137 ^$$$^, 32.5 ^$$$$^)	6 (5, 83.4)	2 (2, 100)	0 (0, 0)	0 (0, 0)
Australia (177)	170 (33, 19.5)	2 (0, 0)	3 (0, 0)	0 (0, 0)	2 (0, 0)
Germany (94)	67 (3, 4.5)	9 (1, 11.1)	8 (2, 25)	7 (1,14.3)	3 (0, 0)
New Zealand (74)	69 (12, 17.4)	3 (0, 0)	2 (0, 0)	0 (0, 0)	0 (0, 0)
France (58)	44 (5, 11.5)	8 (0, 0)	3 (0, 0)	3 (0, 0)	0 (0, 0)
Pakistan (53)	53 (9, 17)	0 (0, 0)	0 (0, 0)	0 (0, 0)	0 (0, 0)
Switzerland (44)	16 (0, 0)	4 (1, 25)	0 (0, 0)	10 (1, 0.1)	14 (1, 7)
Turkey (44)	23 (8, 35)	7 (4, 57)	13 (4, 30.7)	1 (0, 0)	0 (0, 0)
United Kingdom (40)	40 (5, 12.5)	0 (0, 0)	0 (0, 0)	0 (0, 0)	0 (0, 0)
Spain (38)	37 (2, 5.4)	0 (0, 0)	0 (0, 0)	0 (0, 0)	1 (0, 0)
Egypt (24)	22 (5, 22.7)	2 (0, 0)	0 (0, 0)	0 (0, 0)	0 (0, 0)
Saudi Arabia (24)	24 (2, 8.3)	0 (0, 0)	0 (0, 0)	0 (0, 0)	0 (0, 0)
Kyrgyzstan (23)	21 (2, 9.5)	1 (1, 100)	0 (0, 0)	1 (0, 0)	0 (0, 0)
Italy (20)	18 (0, 0)	2 (0, 0)	0 (0, 0)	0 (0, 0)	0 (0, 0)
Lebanon (19)	17 (3, 17.6)	2 (0, 0)	0 (0, 0)	0 (0, 0)	0 (0, 0)
Honk Kong (18)	17 (3, 17.6)	1 (0, 0)	0 (0, 0)	0 (0, 0)	0 (0, 0)
Hungary (17)	8 (3, 37.5)	0 (0, 0)	9 (0, 0)	0 (0, 0)	0 (0, 0)
Iran (16)	14 (5, 35.7)	0 (0, 0)	2 (1, 50)	0 (0, 0)	0 (0, 0)
Yemen (15)	12 (7, 58.4)	1 (0, 0)	0 (0, 0)	0 (0, 0)	0 (0, 0)
Kazakhstan (12)	12 (2, 16.7)	0 (0, 0)	0 (0, 0)	0 (0, 0)	0 (0, 0)
Bulgaria (11)	7 (0, 0)	2 (0, 0)	0 (0, 100)	0 (0, 0)	2 (0, 0)
Ireland (9)	6 (1, 16.7)	1 (0, 0)	1 (0, 0)	1 (0, 0)	0 (0, 0)
Chile (7)	6 (1, 16.7)	1 (0, 0)	0 (0, 0)	0 (0, 0)	0 (0, 0)
United states (6)	5 (0, 0)	1 (0, 0)	0 (0, 0)	0 (0, 0)	0 (0, 0)
Greece (6)	6 (0, 0)	0 (0, 0)	0 (0, 0)	0 (0, 0)	0 (0, 0)
Uzbekistan (5)	4 (0, 0)	1 (0, 0)	0 (0, 0)	0 (0, 0)	0 (0, 0)
Russia Federation (4)	3 (0, 0)	0 (0, 0)	1 (0, 0)	0 (0, 0)	0 (0, 0)
China (4)	4 (0, 0)	0 (0, 0)	0 (0, 0)	0 (0, 0)	0 (0, 0)
United Arab Emirates (4)	4 (0, 0)	0 (0, 0)	0 (0, 0)	0 (0, 0)	0 (0, 0)
Canada (3)	3 (0, 0)	0 (0, 0)	0 (0, 0)	0 (0, 0)	0 (0, 0)
Malaysia (3)	2 (1, 50)	1 (0, 0)	0 (0, 0)	0 (0, 0)	0 (0, 0)
Mexico (3)	3 (1, 33.3)	0 (0, 0)	0 (0, 0)	0 (0, 0)	0 (0, 0)
Ukraine (2)	1 (0, 0)	1 (0, 0)	0 (0, 0)	0 (0, 0)	0 (0, 0)
Belgium (2)	1 (0, 0)	1 (0, 0)	0 (0, 0)	0 (0, 0)	0 (0, 0)
Bosnia (2)	2 (0, 0)	0 (0, 0)	0 (0, 0)	0 (0, 0)	0 (0, 0)
Afghanistan (2)	2 (0, 0)	0 (0, 0)	0 (0, 0)	0 (0, 0)	0 (0, 0)
Mauritius (2)	2 (0, 0)	0 (0, 0)	0 (0, 0)	0 (0, 0)	0 (0, 0)
Armenia (2)	2 (0, 0)	0 (0, 0)	0 (0, 0)	0 (0, 0)	0 (0, 0)
Poland (2)	2 (0, 0)	0 (0, 0)	0 (0, 0)	0 (0, 0)	0 (0, 0)
Oman (2)	2 (0, 0)	0 (0, 0)	0 (0, 0)	0 (0, 0)	0 (0, 0)
Sudan (1)	1 (0, 0)	0 (0, 0)	0 (0, 0)	0 (0, 0)	0 (0, 0)
Nigeria (1)	1 (1, 100)	0 (0, 0)	0 (0, 0)	0 (0, 0)	0 (0, 0)
Indonesia (1)	0 (0, 0)	0 (0, 0)	1 (1, 100)	0 (0, 0)	0 (0, 0)
Korea (1)	0 (0, 0)	1 (0, 0)	0 (0, 0)	0 (0, 0)	0 (0, 0)
Thailand (1)	1 (0, 0)	0 (0, 0)	0 (0, 0)	0 (0, 0)	0 (0, 0)
Kuwait (1)	1 (0, 0)	0 (0, 0)	0 (0, 0)	0 (0, 0)	0 (0, 0)
Morocco (1)	1 (0, 0)	0 (0, 0)	0 (0, 0)	0 (0, 0)	0 (0, 0)
Argentina (1)	1 (0, 0)	0 (0, 0)	0 (0, 0)	0 (0, 0)	0 (0, 0)
Austria (1)	1 (0, 0)	0 (0, 0)	0 (0, 0)	0 (0, 0)	0 (0, 0)
Total	1180 (251, 21)	58 (12, 20.7)	47 (10, 21.3)	25 (2, 8)	20 (1, 5)

^$^ represents the total number of honey samples originating from the mentioned country. ^$$^ represents the total number of samples of each honey type originating from the mentioned country. ^$$$^ represents the rejected samples from the total number of samples from this honey type and ^$$$$^ the percentage of non-conformity out of the total samples from this honey type.

## Data Availability

The data are available from the corresponding author.
